# Theoretical–Computational Study of Atmospheric DBD Plasma and Its Utility for Nanoscale Biocompatible Plasmonic Coating

**DOI:** 10.3390/molecules26165106

**Published:** 2021-08-23

**Authors:** Taj Muhammad Khan, Shahab Ud-Din Khan, Muhammad Raffi, Riaz Khan

**Affiliations:** 1 National Institute of Lasers and Optronics College, Pakistan Institute of Engineering and Applied Sciences, Nilore, Islamabad 45650, Pakistan; muhammad_raffi@hotmail.com; 2 School of Physics and CRANN, Trinity College Dublin, The University of Dublin, Dublin 2, Ireland; 3 Pakistan Tokamak Plasma Research Institute, Nilore, Islamabad 45650, Pakistan; shahab.furqan@gmail.com (S.U.-D.K.); khan.riaz.pk@gmail.com (R.K.)

**Keywords:** atmospheric DBD plasma, plasma simulation, plasma aerosol deposition, antibacterial plasmonic film, high-resolution TEM

## Abstract

In this study, time-dependent, one-dimensional modeling of a surface dielectric barrier discharge (SDBD) device, driven by a sinusoidal voltage of amplitude 1–3 kV at 20 kHz, in argon is described. An SDBD device with two Cu-stripe electrodes, covered by the quartz dielectric and with the discharge gap of 20 × 10^−3^ m, was assumed, and the time-dependent, one-dimensional discharge parameters were simulated versus time across the plasma gap. The plasma device simulated in the given arrangement was constructed and used for biocompatible antibacterial/antimicrobial coating of plasmonic particle aerosol and compared with the coating strategy of the DBD plasma jet. Simulation results showed discharge consists of an electrical breakdown, occurring in each half-cycle of the AC voltage with an electron density of 1.4 × 10^10^ cm^−3^ and electric field strength of 4.5 × 10^5^ Vm^−1^. With SDBD, the surface coating comprises spatially distributed particles of mean size 29 (11) nm, while with argon plasma jet, the nanoparticles are aggregated in clusters that are three times larger in size. Both coatings are crystalline and exhibit plasmonic features in the visible spectral region. It is expected that the particle aerosols are collected under the ionic wind, induced by the plasma electric fields, and it is assumed that this follows the dominant charging mechanisms of ions diffusion. The cold plasma strategy is appealing in a sense; it opens new venues at the nanoscale to deal with biomedical and surgical devices in a flexible processing environment.

## 1. Introduction

The scientific and technological interest in nonthermal plasma arose 70 years back, and since then, plasma technology has become the present and future of manufacturing processes. The development of plasma technology evolved over time, and nowadays, it is used everywhere, in basic commodities to high-tech applications [1,2]. The strategy of nonthermal dielectric barrier discharges (DBDs), in particular, has emerged as a modern young discipline for diverse types of applications, and researchers are aware of its historical roots and impressive inventory. Remarkable developments in DBDs have occurred mostly for economic reasons owing to their easy operation at atmospheric pressure, reliable and energy-efficient operation, flexible adaptation to complex devices, flexible switching between the process conditions, and conventional manufacturing processing.

Briefly, DBD is a nonthermal “cold” plasma in which the electrons have a high temperature, typically 1–5 eV, but the neutral gas and the ions are at about room temperature. It is ignited by applying a high-voltage AC (typically kV at kHz) between two electrodes, wherein at least one of the electrodes is insulated by a dielectric to prevent the occurrence of an arc discharge.

In these plasmas, the electric energy from a power source is transformed mostly to the kinetic energy of the electrons while the rest of the plasma species remain at or close to room temperature, thus consuming a smaller amount of energy. This makes the DBD plasma devices cost-effective for envisioning large-scale future applications.

Over the last couple of decades, DBD plasma technologies have moved into every pore of daily life. In view of enormous leverage, the divulging trend of using these plasmas is obvious from their expanding volume in terms of biomedical, pharmacy, surgical, and biomedical environments, materials synthesis, supported metal catalysts, textiles, surface modification and etching, sterilization, and so forth [1,2,3,4,5,6,7,8,9,10,11,12,13,14,15]. From direct surface sterilization and tailored surface activation to in-line coating deposition of antibacterial/antimicrobial plasmonic active material on surgical tools and medical devices, it has now become one of the paramount needs. Applying the nonthermal plasma at the nanoscale in surgical tools, such as retractors, clamps, sutures, gloves, and imaging devices, has largely assisted surgeons in various processes during the surgery. In pharmacy, DBD plasmas are used for enhancing the flow ability of fine-grained powder with the aim of preventing clogging of the apparatus and precious product losses, and sustaining their longer maintenance times [16]. Titanium surfaces, treated with DBD plasma, are used to inhibit bacterial adhesion and its growth, which cause failure of dental implantation [17]. Progressing and moving the frontier forward, packaging, renewable polymer materials, flexible electronics, biological/biomedical polymers, and PlasmaSkin (dermatological treatments) are now considered the future of plasma science and technology.

DBD plasma devices are configured in different electrode and geometrical arrangements to strike a surface or volumetric discharge [13,14,15]. In DBDs, this discharge is also known as “silent discharge”, and it usually occurs between the two electrodes with one or both the electrodes are covered with a layer of a dielectric material such as quartz, as described in our previous reports [4,15,18]. The dielectric layer is acting as a ballast resistor to the discharge current, preventing arc formation and keeping the plasma in the nonthermal regime. It is also responsible for a self-pulsing operation of the device. Upon ignition, the microdischarges are spatially distributed on the barrier surface and cover a much larger region. The duration of these microdischarges is typically nanosecond and determined by the process gas and discharge arrangement. The planar and cylindrical/tubular geometries are the most common and typical configurations used to produce a diffuse glow or filamentary discharge. In the diffused glow discharge mode, a homogeneous plasma appears, filling the whole space, and the electron charge densities are typically in the range of 10^10^–10^12^ cm^−3^ [10,18,19,20].

Many works have been reported on the simulation of DBD plasmas, operated in the various glow, filamentary, and diffused discharge modes [21,22,23,24]. Gadkari et al. and co-workers [21] modeled a co-axial DBD plasma reactor in pure helium gas using a 2D fluid model in COMSOL Multiphysics and investigated the influence of partial packing on the discharge characteristics. Pan et al. [22] used the fluid model to numerically carry out the evolving features of the atmospheric pressure DBD plasma of tetrafluoromethane (CF_4_). Abidat et al. studied the discharge behavior of DBD plasma, operated in helium and driven by a peak-to-peak voltage of 1 kV at low-frequency 0.2 kHz, with the discharge gap of 3 mm, using the one-dimensional model in COMSOL Multiphysics [23]. Golubovskii et al. [24] used the one-dimensional fluid model and numerically investigated the spatiotemporal characteristics of a homogeneous discharge ignited in helium gas to understand the influence of elementary processes. Similarly, the derivation of electrical discharge in different configurations and working pressures has been reported previously [14]. The 1D model is rather suggested due to the great advantage of reducing the computational time. Studies were conducted on DBD as a process to generate active charge species, ozone, and free radicals for biological and medical applications such as sterilization and decontamination of environmental pollutions [25,26,27]. Processing particle aerosol with the free gas jet has been reported before and interesting results were reported [28]. Processing particle aerosols with the DBD plasma in the surface discharge configuration for biocompatible coating is relatively less reported, though recently, atmospheric plasma jets were employed to entrain the particle aerosol for the deposition on a solid surface, and interesting results were reported [4,15,18].

Here, we report the time-dependent, one-dimensional simulation of DBD plasma strikes in argon gas in the surface barrier discharge mode using double electrodes configuration. The time-dependent various plasma parameters were simulated versus time across the discharge gap. Considering the simulated results, the SDBD plasma device was constructed in the designed geometry, and its utilization for the biocompatible coating of plasmonic nanoparticle aerosols was experimentally demonstrated. Contrary to the chemical precursors used in the chemical plasma deposition, in this novel approach, particle aerosols are formed by the collisional condensation of the ablation plume, which is directly flowed into the plasma active glow discharge region coating. A comparison of the coating produced by the designed SDBD device was made with those produced by argon DBD plasma jet. This study provides a roadmap to the practical realization of SDBD and DBD plasma jet devices for tangible biomedical applications.

## 2. Results and Discussion

### 2.1. Simulation Results

**Figure 1:**
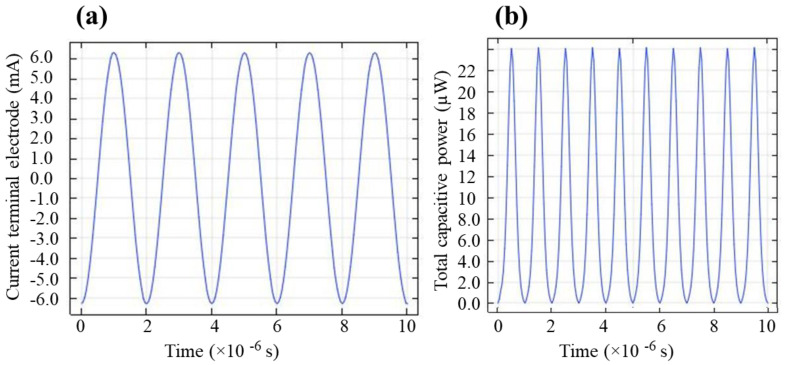
Plasma current of the terminal HV electrode, (**a**) and total capacitive power deposition in the plasma (**b**).

Figure 1a,b respectively describe the temporal evolution of the plasma current (the discharge current flows at the plasma ignition) of the terminal electrode (high-voltage electrode) and total dissipation of the capacitive power of the simulated plasma. When argon plasma is driven by high-power AC voltages, discharge happens and plasma draws a current of an average magnitude of 3.6 × 10^3^ µA in the positive and negative half-cycles of the applied voltage. The gas becomes conductive, and charge accumulates on the surface of the dielectric quartz. The duration of the discharge events in the SDBD is of the order of microsecond and is spatially separated on the same time scale of microsecond. The spatially averaged dissipated power was determined by using the measured transported charge and the voltage drop across the electrodes gap. As expected, the average value of the corresponding power, dissipated in the plasma, is about 13 microwatt (µW), which is significantly small because of the low energy consumption of the plasma device. The low energy consumption makes it cost-efficient, which is desirable for many applications. Although a sufficiently high AC voltage, typically in the kilovolt (kV) range is required to initiate the discharge, the current (μA to mA) is smaller, thus making the power insignificant for the operation of these plasma devices.

**Figure 2:**
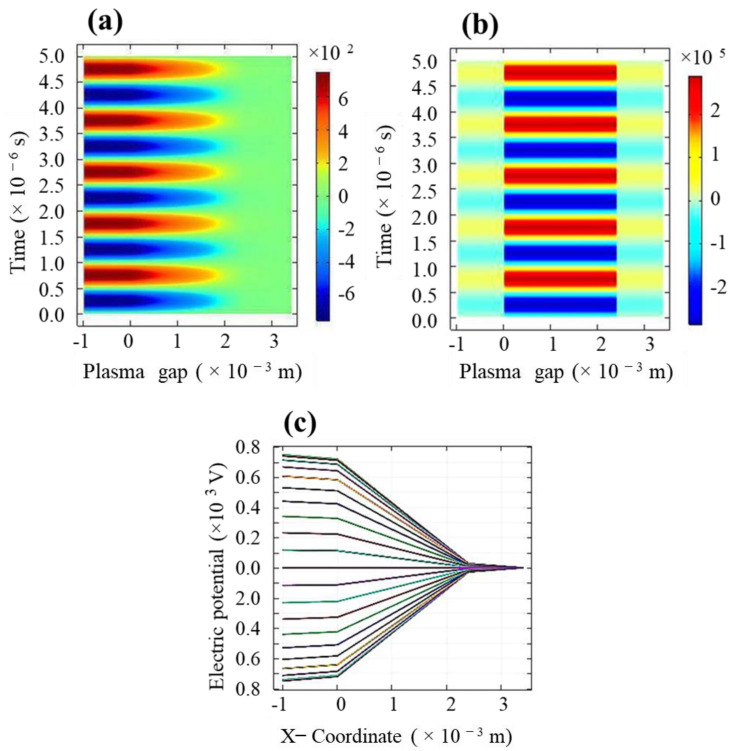
Spatial and temporal evolutions of (**a**) applied electric potential, (**b**) electric field of the plasma as a function of the discharge gap, and (**c**) graphical representation of the electric potential in different times of the voltage cycles.

The evolution of the applied electric potential and electric field across the discharge gap is presented in Figure 2a,b, respectively. The graphical representation of the evolution of the applied potential as a function of the plasma gap in different times is also shown in Figure 2c, where each curve line corresponds to the different times in the range of (17.225–18.094) × 10^−4^ s and different cycles (cycle 2–cycle 10) of the AC voltage. For the designed Cu electrode gap, electric potential in the range of 0.1–0.8 kV is sufficient to sustain discharge in the gas. The electric field established in the plasma gap between the dielectric quartz plates is mitigated since plasma exhibits diamagnetic characteristics. The strength of the simulated electric field is about 4.5 × 10^5^ Vm^−1^. It is noteworthy to mention that, under the existing fields within the plasma, the gas molecules are expected to undergo a momentum transfer from the ions. This expedites the bulk flow of the gas in a process of ionic or electric wind and assists the deposition process.

**Figure 3:**
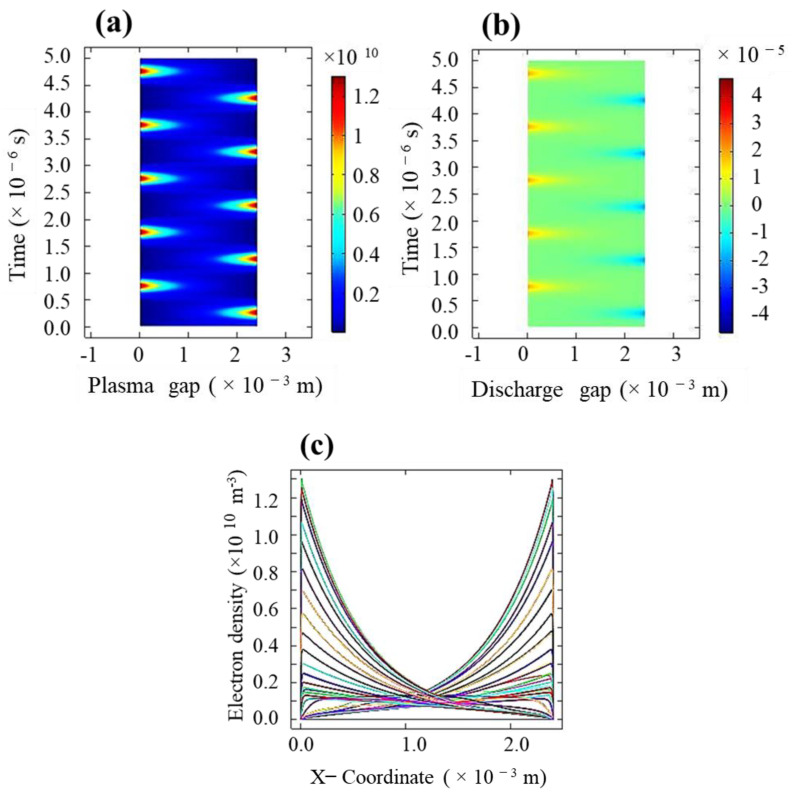
Spatiotemporal evaluations of the electron density (cm^−3^) (**a**) and electron current density (Am^−2^) (**b**), and (**c**) graphical representation of electron density at different times across the discharge gap. Where zero corresponds to the dielectric quartz surface.

Figure 3a,b respectively describe the spatiotemporal evolutions of the electron density and discharge current density in the plasma gap at the instant of gas breakdown in the planner geometry. Clearly, a single discharge occurs per half-cycle of the applied voltage. The event of the first discharge appearing near the live electrode corresponds to the right-hand side of the image. For each discharge, the electron density is maximum at the dielectric surface around the electrode due to the expected glow discharge. Overall, the electron density follows a parabolic distribution with the maximum electron density of about 1.4 × 10^10^ cm^−3^. As the plasma grows up, from the surface towards the center of the plasma gap, the electron density falls off and diminishes for each discharge of the negative and positive cycles of the discharge voltage. The electric potential and electric field change periodically with positions. The value of the simulated average current density of the plasma is about 0.3 × 10^−6^ Am^−2^. Previously, it was reported that the electron density strongly depends on the amplitude and frequency of the AC voltage. It is worth mentioning that the sheath region can be probed using the root mean square value of the electric field [29,30].

**Figure 4:**
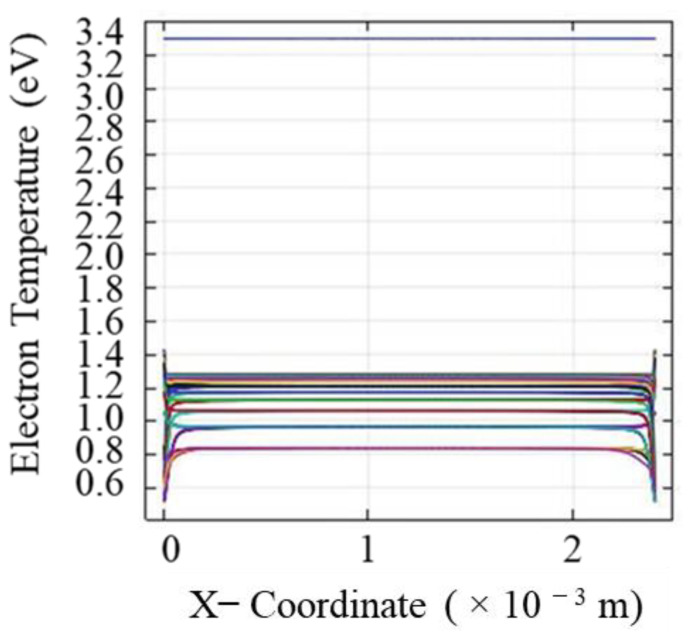
Temperature distribution of electrons across the discharge gap, where zero in the representation figure corresponds to the surface of the dielectric quartz material.

The simulated result of the electron temperature variation across the discharge gap is given in Figure 4, where zero position marks the surface of the dielectric material. The electron temperature grows and diminishes corresponding to each discharge event. Consistent with the reported data in the literature, the distribution of the mean electron temperature corresponding to different times ranges from 0.6–1.3 eV.

### 2.2. Experimetal Results

#### 2.2.1. SDBD Plasma for Antibacterial Plasmonic Aerosols Coating

**Figure 5:**
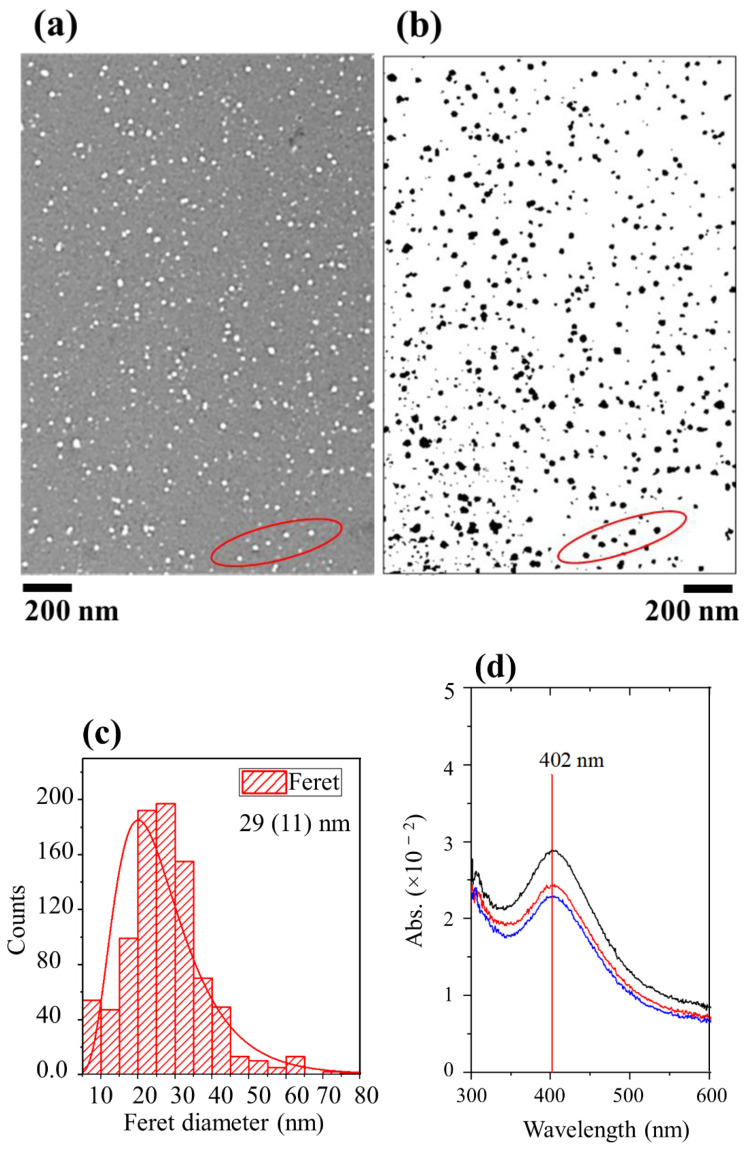
(**a**,**b**) SEM images, (**c**) particle size distribution of the film shown in (**a**) where lognormal function has used, and (**d**) SPR of the film deposited with argon SDBD plasma on Si (**a**) and fused silica (**b**). The SEM image in (**b**) was processed in ImageJ to obtain size distribution. The red circles in both images indicate similar particle regions before and after processing.

**Figure 6:**
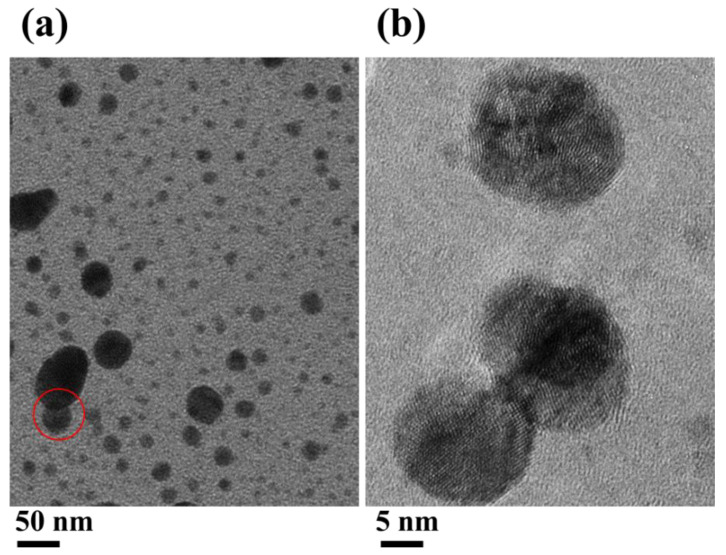
TEM (**a**) and HRTEM (**b**) images of silver NP produced with SDBD plasma and collected on Cu TEM grids covered with an amorphous carbon film. The red circle indicates coupled particles of relatively larger size.

This section describes the deposition of the antibacterial/antimicrobial plasmonic coating of silver done with the arrangement described in the experimental Section 3.1. There are two possible ways of using SDBD plasma for the deposition of layers of a nanomaterial. Using chemical precursors is the most common method applied because the low-energetic ions in these plasmas are excellent reduction agents in nano synthesis and provide a high level of control of the process. Another promising approach is to directly entrain the particle aerosol through the plasma active region for coating without using a chemical precursor. This approach seems effective for biomedical applications since the coating surface and treating objects are placed far beyond the main discharge active region to facilitate the plasma deposition process. Previously, a DBD plasma jet was used, and interesting properties of the coated surface were reported [4,15,18]. The coated surfaces produced were particulate and worked well as a sensitive substrate for the enhanced chemical detection in surface-enhanced Raman spectroscopy (SERS) [18]. The SDBD coated surfaces of Si and fused silica are shown in Figure 5a–d. The image given in (b) has been processed in ImageJ to obtain the particle size distribution displayed in Figure 6c. The areas in the two images, bounded by the red circles, are indicative of similar particle regions before and after image processing. For the given conditions, the surface shows a layered structure of sparsely distributed particles. Overall, there was a slight variation in the particle size and shape and the mean Feret diameter estimated was ~29 (11) nm, where the number in the brackets represents the standard deviation in the size distribution. The particles seem rounded in shape with no pronounced agglomerations. For the given arrangement, the deposition area is relatively smaller; however, the geometry could be modified to obtain a large area of deposition and with high surface coverage. The deposited particles showed surface plasmon resonance (SPR) due to the collective oscillation of the optically excited surface conduction electrons (Figure 5d). The spectra were obtained by scanning three closely spaced regions in the 200–800 nm range. The smaller amplitude of the SPR peak indicates a relatively less populated surface and low coverage. Moreover, the SPR spectrum does not show a long, stretched tail in the infrared region, which signposts that there is no inter-particle dipole–dipole interaction.

The plasma-coated surface was further examined in more detail to catch information on the particle morphology, size, and crystallinity. TEM and HRTEM images captured on the Cu TEM grid, covered with an amorphous carbon film, are displayed in Figure 6a,b, respectively. The surface is mostly covered by a large population of smaller particles (previously not clearly observed in the SEM image) with similar morphology and size. The particles are well isolated from each other except for a couple of particles that are physically connected together, as indicated by the red circle. The coupled particles are of relatively larger size compared with other particles landed on the surface. This effect possibly could come from the strong interaction of the particle with the glow discharge around the electrode. Fringes formation in the TEM high-magnification lattice image designates a crystalline nature of the particle. The elongated large particle is expected from the surface inter-diffusion of the particle aerosols while surging through the glow discharge region. However, further investigation is required to discern the underlying physics and the undergoing mechanisms of interaction of the particle aerosol with the glow discharge and its effect on the particle morphology, size, and distribution. These understandings are potentially significant for advanced biomedical device applications.

**Figure 7:**
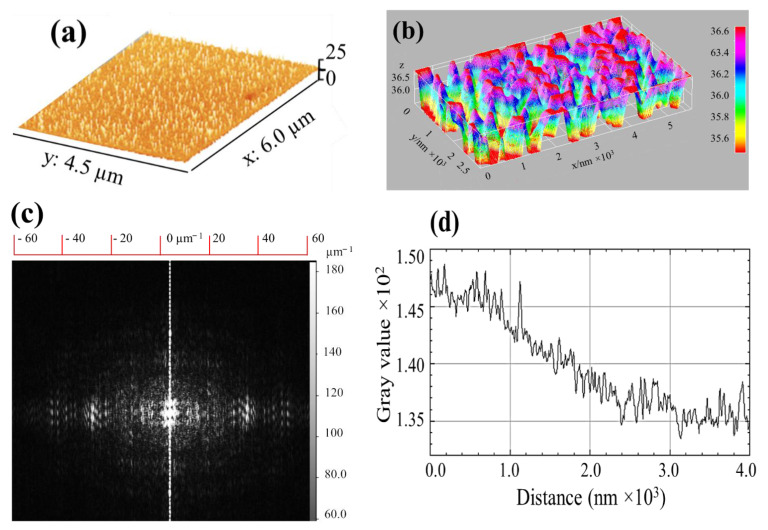
(**a**) Three-dimensional surface morphology, (**b**) 3D interactive surface plot, (**c**) PDS of deposited NP film, and (**d**) surface line profile of the film obtained by ImageJ.

Figure 7a–d provides further information on the morphological features of the plasma-coated surface. Fine features are obvious from the angularly averaged surface plot obtained for a rectangular region on the surface (Figure 7d). Mostly, the surface presents fine and small features along with a fraction of large features, which were also observed in the TEM analysis previously described. The three-dimensional surface morphology and interactive surface plot displayed in Figure 7a,b clearly show particulate features, which are not clearly distinguishable. For statistical analysis of the lateral distribution and homogeneity of the surface structure and topography, a spectral profile called the power spectral density (PSD) was obtained using the 2D fast Fourier transformation (FFT) as shown in Figure 7c. PSD basically gives better interpretation and description of surface geometries, obtained by the FT of the autocorrelation function of the signal, which only contains power across a range of spatial frequencies (wave vectors). A PSD spectral profile was obtained by transforming the image into a frequency domain in which the distribution of the surface height with wavenumbers is described. The bright spots represent the large frequencies area of the real image, while the outer region gives fine details of average roughness. The PSD function in general decomposes the surface features of the original image into wavelengths in imaginary space, which determines the periodicity of the atoms in the reciprocal space. The spatial frequencies (shorter number of wavelengths in R-space) in the PSD clearly represent a good-quality surface without massive irregularities.

It is worth mentioning that the composition, size, and structure of the nanoparticles, as well as agglomerates formation strongly depend on the plasma parameters. These include the plasma energy, electron density, electric field, time, and thermal gradients around each plasma filament as well as the transit time. A limited aspect of these properties has been covered in our early published reports [18]. Broadly speaking, the particle aerosols in the plasma, in principle, follow three charging mechanisms depending upon the dimension of the particle: i) by diffusion of ions (with a particle size <100 nm), ii) by the drift of ions on the field lines intersecting the surface of the particle (coarse particles with size >1 µm), and iii) the combination of field and diffusion charging (particles with size range 100 nm–1 µm) [31,32,33,34]. Obviously, in our case, the latter two mechanisms are ruled out as the particle aerosols are smaller than 100 nm in size, and it is assumed that the dominant mechanism of charging is by the diffusion of ions and this is more prominent for the plasma jet, which is described later. The process of aerosol charging in the plasma active region is interesting as it involves applications such as electrostatic precipitation and coating by electro-collection as well as postdischarge electro-processing for self-repulsion to preserve high interfacial areas, focused deposition or Coulombian coagulation of bipolar aerosols, and measurements based on electrostatic techniques.

#### 2.2.2. Comparison of SDBD and DBD Jet Coating Strategies

**Figure 8:**
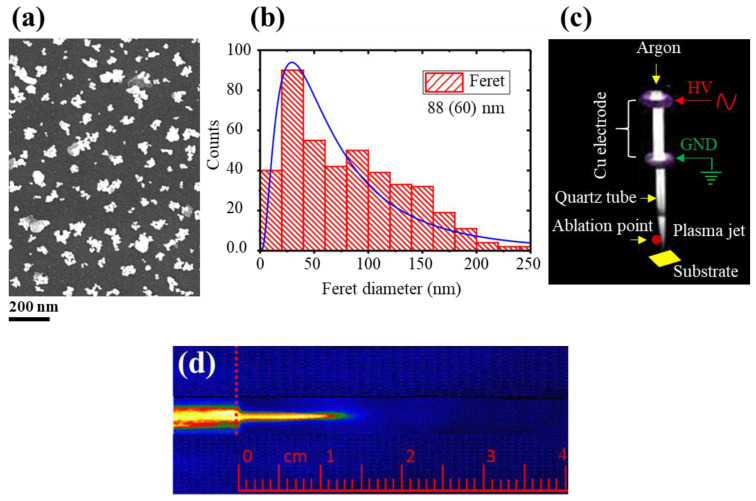
SEM image (**a**) and Feret size distribution (**b**) of silver plasmonic film deposited on Si with argon DBD plasma jet for which the representative iCCD image is shown in (**d**). The DBD plasma jet setup previously used for deposition (**c**). The images have taken from previous work [18] with copy-right permission from Springer Nature.

**Figure 9:**
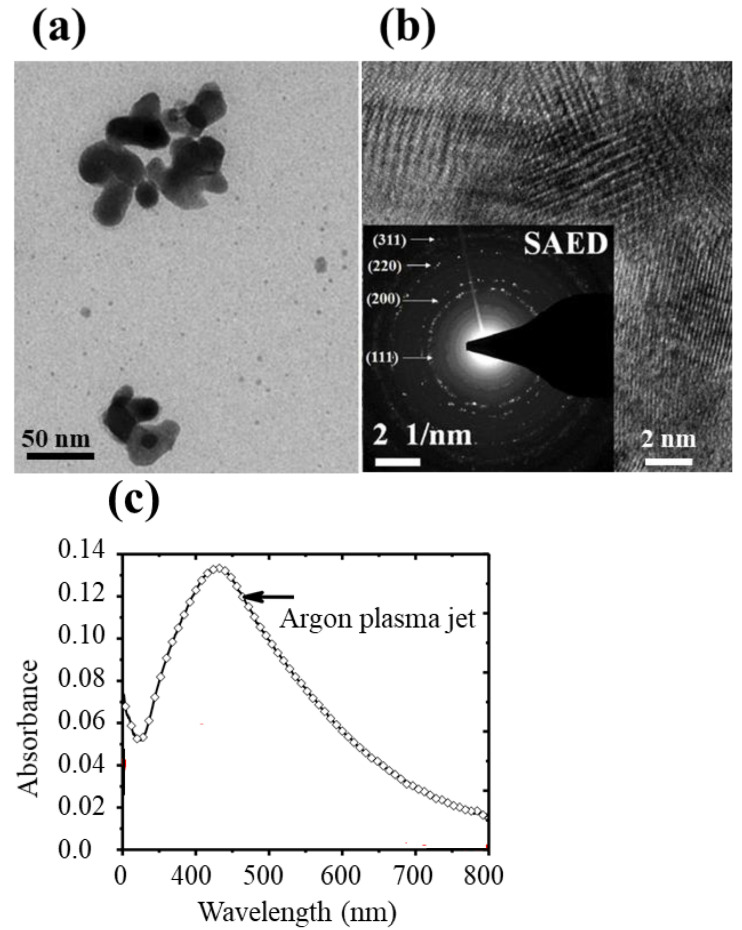
TEM (**a**) and HRTEM (**b**) images of nanoparticle film of silver made with argon DBD plasma jet. SPR absorbance (**c**) of the film shown in Figure 9a. The inset shown in (**b**) is the SAED pattern of the film in (**a**). These images were taken from our previous study [18] with copy-right permission from Springer Nature.

A brief comparison of the antibacterial plasmonic coatings produced in the SDBD arrangement was made with those obtained with the DBD plasma jets. The DBD plasma jet deposition described in Figure 8 has been previously reported [18]. Briefly, DBD plasma devices are potentially capable to launch long luminous plasma effluent, called plasma jets, into the ambient air. The electron charge density (n_e_) and temperature (T_e_) of these typically produced plasma jets are in the range of 10^9^–10^12^ cm^−3^ and 0.2–5 eV, respectively [35,36,37]. For these plasma jets, the plasma bullets propagate at higher speeds in the range of 10^4^–10^6^ ms^−1^, indicating that the process is electrically driven rather than driven by the gas flow [36,37]. The DBD plasma jet setup previously used is also shown in Figure 9c, where the image of the plasma stream displayed in Figure 8d has been taken with an iCCD camera using a longer exposure time (ms) to capture several discharges. As shown in Figure 8a, with nonthermal argon DBD plasma jet, the Si-coated surface is composed of pronounced agglomerations of mean Feret size ~88 (60) nm, where the number in the brackets indicates the standard deviation of the distribution given in Figure 8b. Clearly, there is a striking difference in the morphology, surface features, and particle size of the two cases. With SDBD, the coated particles are well separated and the surface shows single particles with a mean size much smaller than that of the particles obtained with argon DBD plasma jet. In SDBD, the NPs are of pretty uniform sizes and spacing, which may be an indication of the NP charging as described in the previous section. This could happen because a lot of electrons are produced in the plasma which may interact with the metal particle aerosols. The scenario of aerosol deposition is different in the two cases; the plasma jet makes interaction directly with the particle aerosol produced by laser ablation, while in the SDBD device, the particles at first are entrained in the gas and subsequently flowed through the active glow discharge region between the plates for collection on the substrate. This makes feature aspects of the two coatings significantly different from each other.

The aspect of the coating obtained with the plasma jet, examined by TEM and HRTEM (Figure 9a,b), indicated the appearance of large clusters of physically coupled particles. This clearly reveals that the plasma jet strongly modifies the surface morphologies of the particles. In both cases, the particle is crystalline and plasmonic active. In the TEM image, there can be seen smaller particles of mean size 5 (3) nm, sparsely dispersed in the background of the large aggregates. In the case of SDBD, the deposited particles are mostly larger than 10 nm in size. The inset of Figure 9b shows the electron diffraction (SAED) pattern of a region of the coated area and reveals a polycrystalline particle. The observed diffraction rings corresponding to d-spacing values 0.235, 0.206, 0.144, and 0.123 nm can be assigned to the (111), (200), (220), and (311) lattice planes of silver. For the plasma jet coating, the strength of SPR is significantly large (Figure 9c), which indicates a large amount of particulates on the surface. Without going into more detail, briefly, coating with the plasma jets strongly depends upon the jet length, substrate separation, working gas, and gas flowrate [18]. For further understanding, to present a clear picture of the two situations, more work is required in this direction to strongly support the experimental findings. Though, at present, the described work is obviously new and transitory, it is expected that soon this will appear as a promising clean plasma technique with the benefits of sterilization in the antibacterial/antimicrobial coating of biomedical and surgical devices. Furthermore, the underlying study has clearly indicated that the plasma technologies, SDBD and DBD plasma jets, have great potential to open up new possibilities to deposit functional coatings in a continuous system at ambient pressure.

## 3. Materials and Methods

### 3.1. Geometry and Experimental Description

**Figure 10:**
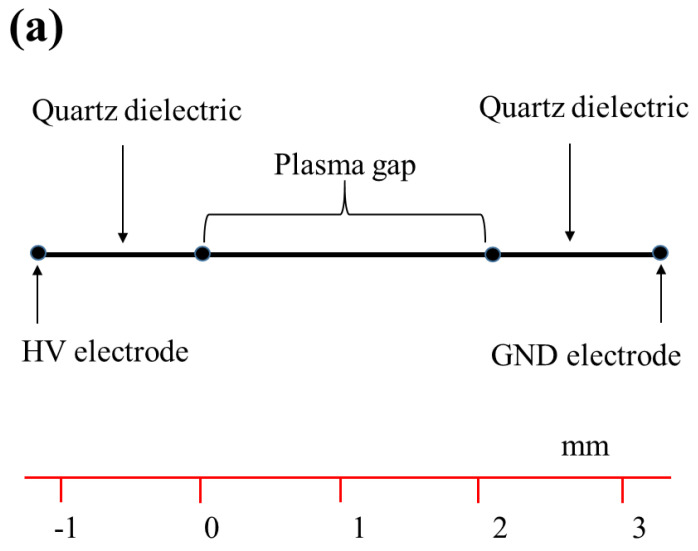

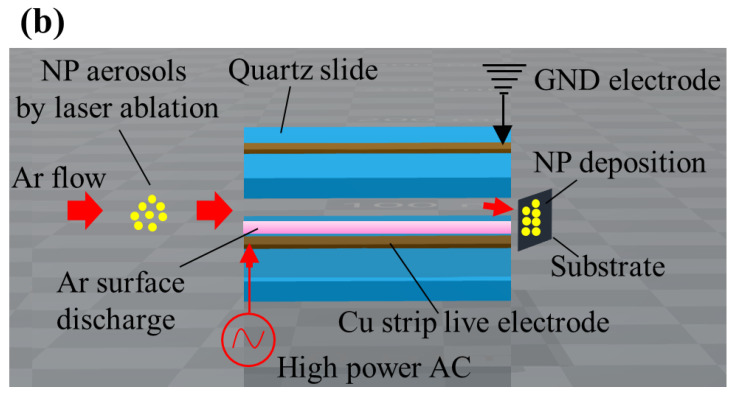
(**a**) One-dimensional geometry used in simulation of COMSOL Multiphysics, and (**b**) geometrical construction of SDBD plasma setup used for coating antibacterial plasmonic nanoparticle aerosols at atmospheric gas pressure.

The one-dimensional geometrical scheme simulated is shown in Figure 10a. The SDBD setup constructed for coating is shown in Figure 10b. The setup shown in **b** consists of two parallel quartz slides, each of thickness 1 × 10^−3^ m, acting as the ballistic resister to limit the discharge current and prevent thermal transitions. The quartz slides are placed 2.4 × 10^−3^ m apart, with two Cu-stripe electrodes of thickness 2.0 × 10^−4^ m, length 1.0 × 10^−2^ m, fixed on the surfaces of the slides with the electrode separation of 20 × 10^−3^ m. One electrode is connected to the HV AC source, and the other electrode is grounded. Argon process gas was supplied at 5.3 ms^−1^ through a capillary tube of inner diameter 2 × 10^−3^ m, and controlled by a mass flow controller. The gas pressure and temperature were 1.0 bar and 300 K, respectively. The surface discharge was driven by using a high-voltage AC of amplitude 1–3 kV at a frequency of 20,000 Hz, and plasma was simulated using COMSOL Multiphysics (version 5.4). Boltzmann equation option was selected to solve a 1D-equation to compute the electron energy distribution function (EEDF). Details of the other input parameters used in simulation are described in Table 1.

Based on the simulation results, the SDBD setup was constructed (Figure 10b), and its utility with the described geometry was experimentally demonstrated in conjunction with the process of laser ablation for biocompatible surface coating of a plasmonic material. The particle aerosols formed by laser ablation were flowed into the active plasma discharge region and deposited on fused silica and polished silicon, used as substrates. Prior to deposition, the substrates were properly cleaned by an ultrasonic cleaner in both alcohol and distilled water. The plasmonic nature, particulate morphology, and crystallinity were examined using UV–Vis spectroscopy, scanning electron microscopy (SEM), transmission electron microscopy (TEM), and high-resolution TEM (HR-TEM). For visualization and analysis of the surface morphology and fine features of the coating, a multiplatform modular Gwyddion software (version 2.59) was used. The distribution of particle Feret size (the longest distance between any two points of the two parallel planes, restricting the particle boundary in a specified direction) was obtained from the SEM image, where a lognormal function was fitted to the distribution to make an estimation of the particle mean size. The lognormal function *f*(*x*) used is given by the expression:

(1) f(x)=A2π×w×xExp ((−ln(xxc)22×w2))

where *A* is the area of the size distribution, *w* the scale parameter defining the width of the size distribution, and *x_c_* the mean radius. The lognormal function fitted to the distribution generated the values of *x_c_* and *w*. The *x_c_* and *w* values were used to calculate the normalized mean size *X_c_* and standard deviation (SD) using the following formulas, previously described in our published report [28].

(2) $$Mean\;size=X_{c}=e^{\ln\left(x_{c}\right)+1/2w^{2}}$$

(3) $$SD=e^{\ln\left(x_{c}\right)+1/2w^{2}}{\left(e^{w^{2}}-1\right)}^{1/2}=X_{c}\times \sqrt{e^{w^{2}}-1}$$

### 3.2. Mathematical Expressions for Simulation

The governing equations of fluid dynamics used in this simulation are described here. Boltzmann equation option was selected to solve the 1D equation to compute the electron energy distribution function (EEDF). The electron density and the electron mean energy were calculated by solving the pair of the propulsion and propagation equations. Mathematical relations describing the discharge dynamics for the electron continuity, and flux are expressed as follows:

(4) $$\frac{\partial n_{e}}{\partial t}+\nabla \cdot {\;\vec{\Gamma }}_{e}=R_{e}-\left(\vec{u}\cdot \nabla \right)n_{e}$$

(5) $$\left(\frac{\partial n_{\varepsilon }}{\partial t}\right)+\nabla \cdot {\;\vec{\Gamma }}_{\varepsilon }+\vec{E}\cdot {\;\vec{\Gamma }}_{\varepsilon }=S_{en}-\left(\vec{u}\cdot \nabla \right)+\left(Q+Q_{gen}\right)$$

where $n_{e}$ is the electron density, $R_{e}$ is the production rate of the electron in the plasma, $\vec{u}$ is the average fluid velocity of the species, ${\;\vec{\Gamma }}_{e}$ is the electron flux, $\vec{E}\cdot {\;\vec{\Gamma }}_{\varepsilon }$ indicates the amount of energy gained by the electron from the electric field, $S_{en}$ is the power dissipation, Q is the net amount of electron charge, and $Q_{gen}$ is the source of heat. 

For the plasma, the time-dependent parameters of temperature and gas absolute pressure were assumed to be 300 K and 1 atm, respectively. The set of initial values of electron density ($n_{e,0}$ = 1 × 10^5^ $m^{-3})$, initial mean electron energy (5 V), electric potential (0 V), refection coefficient ($r_{e}=0$), and thermal emission flux, mean thermionic energy, and electric potential ($V_{0}=V_{rf}V$) were set up for the wall and terminal ($V=V_{0}$). The boundary conditions were set up following equations for the electron flux and energy flux:

(6) $$n\cdot {\;\vec{\Gamma }}_{e}=\frac{1-r_{e}}{1+r_{e}}\left(\frac{1}{2}v_{e,th}n_{e}\right)-\left(\sum^{\;}\gamma_{i}\left({\;\vec{\Gamma }}_{i}\cdot n\right)+{\;\vec{\Gamma }}_{t}\cdot n\right)$$

(7) $$n\cdot {\;\vec{\Gamma }}_{\varepsilon }=\frac{1-r_{e}}{1+r_{e}}\left(\frac{5}{6}v_{e,th}n_{\varepsilon }\right)-\left(\sum^{\;}\gamma_{i}\varepsilon_{i}\left({\;\vec{\Gamma }}_{i}\cdot n\right)+\varepsilon \left({\;\vec{\Gamma }}_{t}\cdot n\right)\right)$$

The second term on the right side of Equation (6) indicates that the electron is produced according to secondary emission, and *γ* is the secondary emission coefficient. For low-incident energies, below 1 eV, the primary electrons tend to be reflected in the plasma boundary wall and the secondary emission coefficient goes to nearly unity. On the surface of the electrodes, ion and excited species become neutralized by the surface reactions. 

Similarly, surface charge accumulation is presented as follows:

(8) $$\frac{\partial \rho_{s}}{\partial t}=n\cdot {\vec{J}}_{i}+n\cdot {\vec{J}}_{e}$$

Here, $n\cdot {\vec{J}}_{i}$ and $n\cdot {\vec{J}}_{e}\;$ are respectively the ion and electron current densities, and $\rho_{s}$ is the surface charge density in the plasma.

## 4. Conclusions and Outlook

The characteristics of a surface dielectric barrier discharge plasma, ignited in argon, were modeled and simulated in COMSOL Multiphysics. Based on simulation, an SDBD setup was constructed, and its utilization in conjunction with the laser ablation process for biocompatible plasmonic film was experimentally demonstrated. The surface discharge coatings so produced were compared with those previously made with an argon DBD plasma jet. The plasma parameters were simulated: the maximum electrons density and temperature were 1.0–1.4 × 10^10^ cm^−3^ and 0.3–0.8 eV, respectively. The designed setup tested for plasmonic active coating worked very well, and films with particle mean size of 29 (11) were produced. The plasma-coated film was crystalline and showed SPR in the visible region in response to optical excitation. It is expected that the particle aerosols are collected and coated under plasma ionic wind following the charging mechanism of ions diffusion, favorable for the particles of mean size less than 100 nm. Contrary to DBD plasma jets, SDBD plasma induced no particles agglomeration. The surface coating made with both plasmas revealed particles of entirely different morphology, sizes, and spatial distribution. It will be of further interest to see the possibility of material deposition within the plasma gap by considering various parameters and increasing the coated area. This work has potential for practical utilization to distribute the created ions over much larger areas, and opens new avenues for further research. Due to the geometrical constraints, it seems good enough to locally treat various surfaces with the SDBD device; however, this is not attractive for the deposition of material on the farthest substrate, which could be fulfilled by employing the coating strategy of DBD plasma jet.

## Figures and Tables

**Table 1:** Input physical and geometrical parameters used to perform simulation and experimental demonstration.

Parameters Used in Simulation Model	
AC Frequency	20 (kHz)
Applied Voltage	−750 [V] × in (w0 × t)
Argon Gas Flowrate	1.0 (lit min^−1^)
Gas Feed SS Tube Dia.	2.0 mm
Electrode Thickness	0.5 (mm)
Dielectric Quartz Thickness	1.0 (mm)
Dielectric Strength of Quartz	25–40 kV/mm
Discharge Gap	2.4 (mm)
Relative Permittivity	10 (ϵ)
Temperature	300 (K)
Absolute Pressure	1.0 (atm)
Argon Density	1.449 (kg/m^3^) (at 300 K)

## Data Availability

Not applicable.

## Sample Availability

Samples are not available from the authors.
